# Supplementary material for: Studying suicide using proxy-based data: reliability and validity of a short version scale for measuring quality of life in rural China
Source: PeerJ. 2021 Nov 9;9:e12396. doi: 10.7717/peerj.12396 (PMC8588864; doi:10.7717/peerj.12396)
Supplement: Supplemental Information 2 [file peerj-09-12396-s002.doc]

**精神卫生与危险意外访谈问卷**

**问卷编号：**________________________ **访谈员姓名：**_________________________

**目标人类别：**1=病例组 2=对照组

**被访人类别：**1=第一信息人 2=第二信息人 0=对照组本人

**目标人姓名：**_________ 生日：______年____月____日（阴历/阳历） 属相______，

年龄：______ 性别：1=男 0=女

**被访人姓名：**_________ 生日：______年____月____日（阴历/阳历） 属相______，

年龄：______ 性别：1=男 0=女

**被访人与目标人的关系：**被访人是目标人的____________

**被访人家庭住址：**________省________（市/县）________（乡/镇）________村，

家庭电话：____________________

**资料收集方式：**1=面对面访谈 2=电话 3=两者并用 4=历史资料

**访谈日期：**__________年__________月__________日

**目标人的居住地区：**1=农村 2=城镇

**访谈地点：**1=被访人的家 2=医院 3=其它地点____________________（请注明）

**访谈全部时间（按24小时制计算）：**

开始时间：__________：__________

结束时间：__________：__________

全部访谈时间：____________________分钟

问卷1

**病例或对照情况**

**目标人基本情况：**

1.他/她（您）的户口所在地： 1=城市户口 2=农村户口

2.他/她（您）的教育水平：上学年数__________年 99=不详

3.最高学历：

1=文盲 2=小学 3=初中 4=高中/中专/高职 5=大专/大学 6=大学以上 88=不适用 99=不详

4.他/她（您）的婚姻状况：

1=单身，从未结婚 2=已婚一起生活 3=已婚但分居

4=再婚 5=离异 6=鳏/寡居

7=未婚同居 88=其它________ 99=不详

5.职业：

1=农民/牧民/渔民 2=个体户 3=干部（公务员/管理人员）

4=工人（包括进城打工者） 5=学生 6=教师

7=乡村医生 8=其他____________（请注明） 99=不详

6.劳动或工作情况：

1=有业/自我雇佣 2=无业 3=退休

88=不适用 99=不详

7.他/她（您）的个人年收入________________元 99=不详

8.他/她（您）的家庭年平均收入：________________元 99=不详

9.他/她（您）的家庭人口（包括本人在内的同住人口）：________人

10.他/她（您）和哪些人一起住？（可多选）

配偶/同居配偶 1

您或您配偶的未成年子女 2

您或您配偶的成年子女 3

您的（外）孙辈 4

您或您配偶的父母 5

其他亲戚 6

其他人 7

独居 8

11. 他/她（您）有子女吗？如果有，一共有多少子女？_______________个（请按照年龄从大到小的顺序记录）

| 关系 | 年龄 | 婚姻状况 | 居住地 | 居住地的原因 | 过去一年回家探望的频率（“回家”是指回父母家） | 过去一年总共住在一起的时间 |
| --- | --- | --- | --- | --- | --- | --- |
|  |  |  |  |  |  |  |
|  |  |  |  |  |  |  |
|  |  |  |  |  |  |  |
|  |  |  |  |  |  |  |
|  |  |  |  |  |  |  |
|  |  |  |  |  |  |  |
|  |  |  |  |  |  |  |
|  |  |  |  |  |  |  |
|  |  |  |  |  |  |  |
|  |  |  |  |  |  |  |

**关系编码**：1. 儿子 2.女儿 3.儿媳 4.女婿

婚姻状况编码：1.已婚 2.离异 3.丧偶 4.分居 5.从未结婚

居住地编码：1.住在一起（一栋楼或者一个院子） 2.本村 3.本乡镇 4. 附近，本县/本市 5.本省 6.外省/外国

居住地原因编码：1.在本地居住 2.出嫁（仅限女儿） 3.定居外地学习/工作（户口已迁出） 4.外出务工（户口仍在本地） 5.其他

过去一年回家探望的频率编码： 1. 住在一起 2.每周1次及以上

3. 1-3次/月 3. 4. 3-11次/年 5. 0-2次/年

总共住在一起时间的编码：1.10-12个月 2.7-9个月 3.3-6个月 4.0-2个月

12.他/她（您）在家庭中的地位如何？

1=最高 2=较高 3=一般 4=低 5=最低 99=不详

13.宗教信仰

1=不信教 2=道教 3=穆斯林 4=基督教 5=天主教 6=佛教

7=其他_____________ 99=不详

14.若他/她（您）有宗教信仰的话，平均每月能参加几次宗教活动？______次/月

15.他/她（您）信不信神（迷信）？ 1=信 0=不信 99=不详

16.他/她（您）信不信人有来世？ 1=信 0=不信 99=不详

17.他/她（您）是共产党员吗？ 1=是 0=否 99=不详

18.他/她（您）的健康状况总的来说属于：

1=非常差 2=比较差 3=一般 4=比较好 5=非常好 99=不详

19.他/她（您）是否曾患有严重或慢性疾病（指曾在医疗机构获得确诊的疾病）？

1=有 0=否 88=不适用 99=不详

**若答案为“否”，请在此标注_ _____，并跳过20—26，直接到27。**

20.若有，依次列出最严重的三种：1=_____________ 2=_____________ 3=_____________

21.这些疾病影响他/她（您）日常生活的程度如何？

1=无影响 2=轻度影响 3=中度影响 4=深度影响 99=不详

22.这些疾病影响他/她（您）精神状况的程度如何？

1=无影响 2=轻度影响 3=中度影响 4=深度影响 99=不详

23. 他/她（您）有没有说过这些疾病给他/她（您）的生命带来威胁或者提到这个病活不了多久？

1.有 2.无 99.不详

24. 他/她（您）有没有说过这些病给家庭带来经济负担？如果有，有多严重？

1.完全无负担 2.比较轻的负担 3.比较严重的负担 4.很重的负担

25．他/她（您）有没有说过因为这些疾病需要照顾，给家人带来身体和精神上的负担？

1.完全无负担 2.比较轻的负担 3.比较严重的负担 4.很重的负担

26. 他/她（您）在出事前（最近）有没有感到疼痛（包括各种躯体疾病导致的疼痛或不明原因的疼痛）？如果有，可以评几分？0是完全不疼痛，10是最严重的无法忍受的疼痛。

|  |  |  |  |  |  |  |  |  |  |
| --- | --- | --- | --- | --- | --- | --- | --- | --- | --- |

0 1 2 3 4 5 6 7 8 9 10

27.他/她（您）有精神疾病吗？ 1=有 0=无 99=不详

28.若“有”，他/她（您）得到治疗了吗？ 1=有 0=否 99=不详

29.他/她（您）的家庭成员**（注意：指有血缘关系的）**有曾经自杀**（注意：包括自杀未遂和自杀死亡）**的吗？

1=有 0=无 99=不详

若有，此家庭成员为__________________________；其自杀方式为____________________

30. 他/她（您）的其他**无血缘关系**的家庭成员或熟人中，有曾经自杀**（注意：包括自杀未遂和自杀死亡）**吗？

1=有 0=无 99=不详

若有，此人为__________________________；其自杀方式为____________________

31.他/她（您）家中是否储藏有农药？ 1=是 0=否 99=不详

如果是，请列出三种主要农药名称：

1_______________ 2_______________ 3_______________

**32—36适用于自杀死亡组目标人、对照组目标人。若为对照组目标人，则医院（诊所）为离对照组目标人家最近的那所医院（诊所），距离为其家到那所医院（诊所）的距离。**

32.他/她出事后被送（离您家最近的）______________________医院（诊所）；位置____________________________; 其级别为：_______________级

33.他/她（您）家所在村庄的正式名字：_______________。

34.从事发地（您家）到医院的估计距离：__________里。

35.从事发地（您家）到医院需花费的时间：__________小时。

36.出事前/最近的一周或一月内，他/她是否看过医生？

1=是 0=否 99=不详

|  | 最近一周 | 最近一月 |
| --- | --- | --- |
| 中医 |  |  |
| 精神科医生或其他心理卫生工作者 |  |  |
| 村医 |  |  |
| 乡镇卫生院的其他科医生 |  |  |
| 县级或县级以上医院的其他科医生 |  |  |

问卷2

**信息人半定式访谈提纲（注意：仅用于自杀组）**

1. 您认为他/她自杀的原因是什么？

（请尽可能全面。如未提及，请注意补充以下方面的信息：躯体疾病、心理健康、家庭关系、与社区和邻里的关系、成为家庭/他人/社会的负担、经济困难）

1. 他/她的自杀对您有什么样的影响？

问卷3

**自杀行为情况**

现在我要问您一些与他/她（试图）自杀以及触发这一事件发生有关的详细情况，请尽可能详细回忆细节。（**注：若目标人是对照组目标人，则直接跳到问题21。**）

1.他/她自杀日期（非死亡时间）：________年______月______日

2.他/她自杀发生的准确时间（以24小时方式记录）：______时______分

3.自杀者首先使用的是哪一种方法：__________

1=喝农药 2=喝其他毒药 3=上吊 4=投河 5=跳楼 6=过量服药 7=割腕

8=煤气 9=窒息 10=开枪 11=触电 12=卧轨 13=其他________

88=不适用 99=不详

4.导致自杀者死亡的方法是：____________________

5.他/她自杀的地点在 1=家中 2=其他地方________________________

6.他/她自杀前内6小时内是否有喝酒？ 1=是 0=否 99=不详

若选“是”：

（1）他/她是在自杀前多长时间喝酒的？___________________小时

（2）他/她在自杀前喝了多少“杯”？ （一两高度白酒=一瓶啤酒=2杯；如果是私酿酒，如米酒或高粱酒，请记录度数和量）

__________________杯 **或** ___________两___________度的酒

7.事情发生时，他/她在什么地方？其他什么人在场或在附近？

（SIS-1）隔离性

0=有人在场（指面对面）

1=有人在附近，能看得见或听得见

2=附近无人，或无人看见或听见

8.为防止被发现，他/她做过什么？（如有无锁门、选择不熟悉的地方、有线索和暗示）谁发现了他/她？ 怎样发现的？

（SIS-2）从时间上

0=可能被阻止

1=不大可能被阻止

2=完全不可能被阻止

9.（SIS-3）防范（释：事先做好准备以防止被发现的行为）

0=无防范

1=被动防范（如躲避别人，但不防止别人干预；独自在房间门不上锁）

2=主动防范（如锁门）

10.（SIS-4）寻求帮助

0=向可能帮助自己者告诉了自杀企图

1=与可能帮助自己者联系过但未明确表明自杀企图

2=未与可能帮助自己者联系过或告知自杀企图

11.他/她是否为死亡作了什么准备？（如了却心愿或将对自己有特殊意义的物品送人？）

（SIS-5）后事安排

0=没有

1=考虑过或作出过一些安排

2=做出了确定的计划或完成了安排

12.他/她对自杀做过多少计划？（如割脉或寻找毒药、储存药片等）

（SIS-6）积极准备

0=无准备

1=部分/轻度到中等准备

2=完全/充分准备

13.他/她是否写过遗言或遗书，或者把自杀计划写进日记？他/她是否打算这样做？

（SIS-7）遗言、遗书或有关日记

0=没有

1=写了遗言、遗书但又撕碎了，或日记中提到但又划掉了；考虑过自杀的遗言、信件或日记

2=有遗言、遗书或有关日记

14.除了留言，他/她将自己的计划告诉了谁？所说的内容是什么？____________是否曾经说过什么，使别人回想起的时候会说“我早就应该知道他会自杀”？

（SIS-8）行动前明确与人提到自杀企图

0=没有

1=模模糊糊地提及

2=明确地提及

15.自杀后，谁最先发现他/她的？

1=家庭成员 2=亲属 3=朋友 4=其他 99=不详

16.发现他/她自杀后，目击者第一反应是什么？

1=拨打急救电话 2=告诉目标人的家人 3=打电话叫医生

4=其他 99=不详

17.他/她被发现之后，是否立即被送往医院？

1=是 0=否 99=不详

18.事件发生后，有人马上对他/她实施抢救吗？

1=有 0=无 99=不详

19.他/她被发现后多长时间才得到有效的治疗？ _______________小时

20.您认为触发他/她发生自杀的直接原因是：_________________________

21.他/她（您）曾经试图自杀过吗？过去所有试图自杀的次数：____________

**若答案为0次，请直接到下一问卷。**

22.上次试图自杀离该事件发生（现在）有多长时间？

1=1~7天 2=2~4个星期 3=2~6个月 4=7~12个月 5=2年

6=3~5年 7=6年及以上 88=不适用 99=不详

23.上次试图自杀的方法为：

1=喝农药 2=喝其他毒药 3=上吊 4=投河 5=跳楼 6=过量服药

7=割腕 8=煤气 9=窒息 10=开枪 11=触电 12=卧轨

13=其它________ 88=不适用 99=不详

问卷4

**BIS冲动量表中文版**

在不同的情况下，人们的行为和思维也不一样。这是一个测量您的一些行为和思维方式的量表，请仔细阅读并快速如实地回答。

| 1. 我认真安排每件事。 | 不是 | 极少 | 有时 | 经常 | 总是 |
| --- | --- | --- | --- | --- | --- |
| 1. 我做事不加思考。 | 不是 | 极少 | 有时 | 经常 | 总是 |
| 1. 遇到问题时我能想出好办法。 | 不是 | 极少 | 有时 | 经常 | 总是 |
| 1. 我对未来有计划。 | 不是 | 极少 | 有时 | 经常 | 总是 |
| 1. 我不能很好地控制自己的行为。 | 不是 | 极少 | 有时 | 经常 | 总是 |
| 1. 必要时我能够长时间考虑一个问题。 | 不是 | 极少 | 有时 | 经常 | 总是 |
| 1. 我有规律地存钱或攒钱。 | 不是 | 极少 | 有时 | 经常 | 总是 |
| 1. 我难以控制自己的脾气。 | 不是 | 极少 | 有时 | 经常 | 总是 |
| 1. 我能从不同的角度考虑问题。 | 不是 | 极少 | 有时 | 经常 | 总是 |
| 1. 我对工作和获得收入有计划。 | 不是 | 极少 | 有时 | 经常 | 总是 |
| 1. 我说话不加思考。 | 不是 | 极少 | 有时 | 经常 | 总是 |
| 1. 遇到问题时我喜欢慢慢考虑。 | 不是 | 极少 | 有时 | 经常 | 总是 |
| 1. 我做事比较理智。 | 不是 | 极少 | 有时 | 经常 | 总是 |
| 1. 我激动时难以控制自己的行为。 | 不是 | 极少 | 有时 | 经常 | 总是 |
| 1. 遇到难题时我可以耐心地思考解决问题的办法。 | 不是 | 极少 | 有时 | 经常 | 总是 |
| 1. 我有规律地安排饮食起居。 | 不是 | 极少 | 有时 | 经常 | 总是 |
| 1. 我容易冲动行事。 | 不是 | 极少 | 有时 | 经常 | 总是 |
| 1. 做决定前，我喜欢仔细考虑得失。 | 不是 | 极少 | 有时 | 经常 | 总是 |
| 1. 我离开家之前把事情都安排好。 | 不是 | 极少 | 有时 | 经常 | 总是 |
| 1. 我不考虑后果而立即行动。 | 不是 | 极少 | 有时 | 经常 | 总是 |
| 1. 我冷静地思考问题。 | 不是 | 极少 | 有时 | 经常 | 总是 |
| 1. 我做事时能按计划完成。 | 不是 | 极少 | 有时 | 经常 | 总是 |
| 1. 我容易冲动性购物。 | 不是 | 极少 | 有时 | 经常 | 总是 |
| 1. 遇到难题时我不会轻易下结论。 | 不是 | 极少 | 有时 | 经常 | 总是 |
| 1. 我花钱有计划性。 | 不是 | 极少 | 有时 | 经常 | 总是 |
| 1. 我做事十分莽撞。 | 不是 | 极少 | 有时 | 经常 | 总是 |
| 1. 我思考问题时能集中注意力。 | 不是 | 极少 | 有时 | 经常 | 总是 |
| 1. 我很看重对未来的安排。 | 不是 | 极少 | 有时 | 经常 | 总是 |
| 1. 我想到什么就马上去做。 | 不是 | 极少 | 有时 | 经常 | 总是 |
| 1. 我容易想出新的办法来解决遇到的困难。 | 不是 | 极少 | 有时 | 经常 | 总是 |

问卷5 **绝望量表**

指导语：

每个人对未来都有一些看法，目标人的看法如何？以下每个问题都有5个答案：1.完全符合 2.基本符合 3.难以确定 4.基本相反 5.完全相反

请根据目标人**自杀前一周**（访谈前一周）的情况，选择一个最符合的答案。

|  | 完全  符合 | 基本  符合 | 难以  确定 | 基本  相反 | 完全相反 |
| --- | --- | --- | --- | --- | --- |
| 1.他/她（您）希望将来能把最紧要的事情做好。 | 1 | 2 | 3 | 4 | 5 |
| 2.他/她（您）的未来一片黑暗。 | 1 | 2 | 3 | 4 | 5 |
| 3.他/她（您）运气不好，也不指望以后会时来运转。 | 1 | 2 | 3 | 4 | 5 |
| 4.他/她（您）对未来充满信心。 | 1 | 2 | 3 | 4 | 5 |

**问卷6 生活质量**

下面我想了解您最近一个月内的生活质量。

| 近一个月内您的身体状况怎样？ | 非常好 | 好 | 一般 | 差 | 非常差 |
| --- | --- | --- | --- | --- | --- |
| 近一个月内您的精神心理状况怎样 | 非常好 | 好 | 一般 | 差 | 非常差 |
| 近一个月内您的经济状况怎样？ | 非常好 | 好 | 一般 | 差 | 非常差 |
| 近一个月内您的工作（学习、做农活）状况怎样？ | 非常好 | 好 | 一般 | 差 | 非常差 |
| 近一个月内您与家人的关系怎样？ | 非常好 | 好 | 一般 | 差 | 非常差 |
| 近一个月内您与其他人的关系怎样？ | 非常好 | 好 | 一般 | 差 | 非常差 |

问卷7

**老年人生活事件量表**

指导语：下表中列出了生活中可能会发生的事件。每个事件都有五个栏目，分别为事情发生的时间、性质、对心理影响程度、影响持续时间和事件发生次数。请在每个栏目中选择最恰当的一项划“√”。若该事件未发生，只在“未发生”一项下划“√”，其余四栏不填。

| **生 活 事 件** | **发生**  **时间** | | | **性质** | | **心理影**  **响程度** | | | | | **影响持**  **续时间** | | | | **发生次数** |
| --- | --- | --- | --- | --- | --- | --- | --- | --- | --- | --- | --- | --- | --- | --- | --- |
|  | 未发生 | 一年内 | 长期性 | 好  事 | 坏  事 | 无影响 | 轻  度 | 中  度 | 重  度 | 极  重 | 三月内 | 半年内 | 一年内 | 一年以上 |  |
| 举例：搬家 |  | √ |  |  | √ |  | √ |  |  |  |  | √ |  |  | 1 |
| 健康有关问题 |  |  |  |  |  |  |  |  |  |  |  |  |  |  |  |
| 1.患有慢性疾病 |  |  |  |  |  |  |  |  |  |  |  |  |  |  |  |
| 2.本人重病或得绝症 |  |  |  |  |  |  |  |  |  |  |  |  |  |  |  |
| 3.家庭成员重病或得绝症 |  |  |  |  |  |  |  |  |  |  |  |  |  |  |  |
| 4.本人因交通事故或其他意外受伤 |  |  |  |  |  |  |  |  |  |  |  |  |  |  |  |
| 5.家庭成员因交通事故或其他意外受伤 |  |  |  |  |  |  |  |  |  |  |  |  |  |  |  |
| 6.本人住院治疗 |  |  |  |  |  |  |  |  |  |  |  |  |  |  |  |
| 7.家庭成员住院治疗 |  |  |  |  |  |  |  |  |  |  |  |  |  |  |  |
| 8.本人自理生活困难 |  |  |  |  |  |  |  |  |  |  |  |  |  |  |  |
| 9.家庭成员自理生活困难 |  |  |  |  |  |  |  |  |  |  |  |  |  |  |  |
| 10.本人从疾病中康复 |  |  |  |  |  |  |  |  |  |  |  |  |  |  |  |
| 11.家庭成员从疾病中康复 |  |  |  |  |  |  |  |  |  |  |  |  |  |  |  |
| 12.亲戚或好友重病 |  |  |  |  |  |  |  |  |  |  |  |  |  |  |  |
| 13.配偶死亡 |  |  |  |  |  |  |  |  |  |  |  |  |  |  |  |
| 14.子女死亡 |  |  |  |  |  |  |  |  |  |  |  |  |  |  |  |
| 15.子女的配偶死亡 |  |  |  |  |  |  |  |  |  |  |  |  |  |  |  |
| 16.亲戚或好友死亡 |  |  |  |  |  |  |  |  |  |  |  |  |  |  |  |
| 家庭生活有关问题： |  |  |  |  |  |  |  |  |  |  |  |  |  |  |  |
| 17.与配偶有激烈争吵或打架 |  |  |  |  |  |  |  |  |  |  |  |  |  |  |  |
| 18.夫妻分居 |  |  |  |  |  |  |  |  |  |  |  |  |  |  |  |
| 19.离婚 |  |  |  |  |  |  |  |  |  |  |  |  |  |  |  |
| 20.本人有外遇 |  |  |  |  |  |  |  |  |  |  |  |  |  |  |  |
| 21.配偶有外遇 |  |  |  |  |  |  |  |  |  |  |  |  |  |  |  |
| 22.夫妻重归于好 |  |  |  |  |  |  |  |  |  |  |  |  |  |  |  |
| 23.子女与其配偶有激烈争吵 |  |  |  |  |  |  |  |  |  |  |  |  |  |  |  |
| 24.家庭经济困难 |  |  |  |  |  |  |  |  |  |  |  |  |  |  |  |
| 25.独居 |  |  |  |  |  |  |  |  |  |  |  |  |  |  |  |
| 26.住房拥挤 |  |  |  |  |  |  |  |  |  |  |  |  |  |  |  |
| 27.失窃或房屋、财产的重大损失 |  |  |  |  |  |  |  |  |  |  |  |  |  |  |  |
| 28.子女长期离家 |  |  |  |  |  |  |  |  |  |  |  |  |  |  |  |
| 29.子女不孝 |  |  |  |  |  |  |  |  |  |  |  |  |  |  |  |
| 30.饮食或睡眠习惯的较大改变 |  |  |  |  |  |  |  |  |  |  |  |  |  |  |  |
| 31.家庭成员之间关系不和 |  |  |  |  |  |  |  |  |  |  |  |  |  |  |  |
| 32.子女下岗待业或就业困难 |  |  |  |  |  |  |  |  |  |  |  |  |  |  |  |
| 33.个人居住或生活条件有较大改变 |  |  |  |  |  |  |  |  |  |  |  |  |  |  |  |
| 34.经济情况显著改善 |  |  |  |  |  |  |  |  |  |  |  |  |  |  |  |
| 社交及其他问题 |  |  |  |  |  |  |  |  |  |  |  |  |  |  |  |
| 35.与邻居关系紧张或发生争执 |  |  |  |  |  |  |  |  |  |  |  |  |  |  |  |
| 36.与好友分离或决裂 |  |  |  |  |  |  |  |  |  |  |  |  |  |  |  |
| 37.没有知心朋友、孤独 |  |  |  |  |  |  |  |  |  |  |  |  |  |  |  |
| 38.本人离职退休 |  |  |  |  |  |  |  |  |  |  |  |  |  |  |  |
| 39.配有离职退休 |  |  |  |  |  |  |  |  |  |  |  |  |  |  |  |
| 40.本人卷入法律纠纷 |  |  |  |  |  |  |  |  |  |  |  |  |  |  |  |
| 41.家庭成员卷入法律纠纷 |  |  |  |  |  |  |  |  |  |  |  |  |  |  |  |
| 42.本人遭遇丢面子、受人歧视 |  |  |  |  |  |  |  |  |  |  |  |  |  |  |  |
| 43.被人误会或错怪 |  |  |  |  |  |  |  |  |  |  |  |  |  |  |  |
| 44.本人遭遇他人恐吓、殴打 |  |  |  |  |  |  |  |  |  |  |  |  |  |  |  |
| 45.家庭成员遭遇他人恐吓、殴打 |  |  |  |  |  |  |  |  |  |  |  |  |  |  |  |
| 46.被他人欺骗 |  |  |  |  |  |  |  |  |  |  |  |  |  |  |  |
| 如果最近一年内您还经历其他生活事件，请填写 |  |  |  |  |  |  |  |  |  |  |  |  |  |  |  |
|  |  |  |  |  |  |  |  |  |  |  |  |  |  |  |  |
|  |  |  |  |  |  |  |  |  |  |  |  |  |  |  |  |

问卷8

**社会支持量表（**DSSI**）**

**社会交往分量表**

1.除家庭成员外，在本地区一个小时可及的范围内，他/她说过（您觉得）有多少人可以依赖或感到很亲近？

1=0 2=1~4人 3=5人及以上

2.除工作外，他/她在自杀前一周（您在上一周）内有多少次与不住在一起的人有来往：包括他/她去看别人、别人去看他/她或他们（您们）一起出门。

1=0 2=1~4人 3=5人及以上

3.除工作外，他/她在自杀前一周（您在上一周）内有多少次在电话上与朋友、亲戚或其他人谈话（电话打给他/她或他/她给别人打都计算）。

1=0 2=1~4次 3=5次及以上

4.除工作外，他/她在自杀前一周（您在上一周）内有多少次参加各种聚会。

1=0 2=1~4次 3=5次及以上

**主观社会支持**

5.他/她是否说过他/她的家人和朋友（即对他/她重要的人）理解他/她？

1=几乎从未说过 2=有时这样说 3=经常这样说

6.他/她是否说过他/她对家人和朋友（即对他/她重要的人）是有用的？

1=几乎从未说过 2=有时这样说 3=经常这样说

7.他/她是否说过他/她知道家人和朋友的近况？

1=几乎从未说过 2=有时这样说 3=经常这样说

8.他/她是否说过他/她感到与家人和朋友在谈话时别人在听他/她？

1=几乎从未说过 2=有时这样说 3=经常这样说

9.他/她是否说过他/她在家人和朋友中间有确定的位置（角色）？

1=几乎从未说过 2=有时这样说 3=经常这样说

10.他/她是否说过他/她至少可以同有些家人和朋友谈心里话？

1=几乎从未说过 2=有时这样说 3=经常这样说

11.他/她是否说过他/她对与家人和朋友关系有多满意？如果没有家人和朋友，他/她对没有这种关系是否感到满意？

1=十分不满意 2=有所满意 3=满意

**社会支持利用分量表**

现在我要问一些他/她觉察到的家人和朋友帮助他/她的方式。他/她是否说过他/她的家人曾用以下方式帮助他/她？

12.在他/她生病的时候帮助他/她？

1=是 0=否

13.帮他/她购物或跑腿？

1=是 0=否

14.给他/她礼物？

1=是 0=否

15.花钱给他/她帮助？

1=是 0=否

16.帮他/她整理房屋周围环境？

1=是 0=否

17.帮他/她整理房屋，料理家务？

1=是 0=否

18.为他/她的生意或经济问题提供建议？

1=是 0=否

19.陪伴他/她？

1=是 0=否

20.听他/她诉苦？

1=是 0=否

21.为他/她如何处理生活问题提供建议？

1=是 0=否

22.为他/她提供交通方便？

1=是 0=否

23.为他/她做饭或请他/她吃饭？

1=是 0=否

问卷9

**老年抑郁量表**

注：请选择最符合您**过去一周**中感受的答案。在调查员念完一句话之后，以“是”或“否”作答。

| 1、您对生活基本上满意吗？ | 是 | 否 |
| --- | --- | --- |
| 2、您是否已放弃了许多活动与兴趣？ | 是 | 否 |
| 3、您是否觉得生活空虚？ | 是 | 否 |
| 4、您是否感到厌倦？ | 是 | 否 |
| 5、您觉得未来有希望吗？ | 是 | 否 |
| 6、您是否因为脑子里一些想法摆脱不掉而烦恼？ | 是 | 否 |
| 7、您是否大部分时间精力充沛？ | 是 | 否 |
| 8、您是否害怕会有不幸的事落到你头上？ | 是 | 否 |
| 9、您是否大部分时间感到幸福？ | 是 | 否 |
| 10、您是否常感到孤立无援？ | 是 | 否 |
| 11、您是否经常坐立不安，心烦意乱？ | 是 | 否 |
| 12、您是否愿意呆在家里而不愿去做些新鲜事？ | 是 | 否 |
| 13、您是否常常担心将来？ | 是 | 否 |
| 14、您是否觉得记忆力比以前差？ | 是 | 否 |
| 15、您觉得现在活着很惬意吗？ | 是 | 否 |
| 16、您是否常感到心情沉重、郁闷？ | 是 | 否 |
| 17、您是否觉得像现在这样活着毫无意义？ | 是 | 否 |
| 18、您是否总为过去的事忧愁？ | 是 | 否 |
| 19、您觉得生活很令人兴奋吗？ | 是 | 否 |
| 20、您开始一件新的工作很困难吗？ | 是 | 否 |
| 21、您觉得生活充满活力吗？ | 是 | 否 |
| 22、您是否觉得您的处境已毫无希望？ | 是 | 否 |
| 23、您是否觉得大多数人比您强得多？ | 是 | 否 |
| 24、您是否常为些小事伤心？ | 是 | 否 |
| 25、您是否常觉得想哭？ | 是 | 否 |
| 26、您集中精力有困难吗？ | 是 | 否 |
| 27、您早晨起来很快活吗？ | 是 | 否 |
| 28、您希望避开聚会吗？ | 是 | 否 |
| 29、您做决定很容易吗？ | 是 | 否 |
| 30、您的头脑像往常一样清晰吗？ | 是 | 否 |

问卷10  **家庭功能问卷**

| 1. 当我遇到困难时，可以得到家人满意的帮助 | 经常 | 有时 | 很少 |
| --- | --- | --- | --- |
| 1. 我很满意家人与我讨论各种事情以及分担问题的方式 | 经常 | 有时 | 很少 |
| 1. 我做的事情，家人都能接受且给予支持 | 经常 | 有时 | 很少 |
| 1. 我很满意家人相互尊重、接受、重视等感情的表达 | 经常 | 有时 | 很少 |
| 1. 我很满意家人与我共度时光（在一起）的方式（旅游、娱乐等） | 经常 | 有时 | 很少 |

问卷11

**日常生活活动能力量表**

| **测试类别** | **评 分** |
| --- | --- |
| D1使用公共车辆 | 1自己完全可以做 2有些困难 3需要帮助 4根本无法做 |
| D2行走 | 1自己完全可以做 2有些困难 3需要帮助 4根本无法做 |
| D3做饭菜 | 1自己完全可以做 2有些困难 3需要帮助 4根本无法做 |
| D4做家务 | 1自己完全可以做 2有些困难 3需要帮助 4根本无法做 |
| D5吃药 | 1自己完全可以做 2有些困难 3需要帮助 4根本无法做 |
| D6吃饭 | 1自己完全可以做 2有些困难 3需要帮助 4根本无法做 |
| D7穿衣 | 1自己完全可以做 2有些困难 3需要帮助 4根本无法做 |
| D8梳头、刷牙等 | 1自己完全可以做 2有些困难 3需要帮助 4根本无法做 |
| D9洗衣 | 1自己完全可以做 2有些困难 3需要帮助 4根本无法做 |
| D10洗澡 | 1自己完全可以做 2有些困难 3需要帮助 4根本无法做 |
| D11购物 | 1自己完全可以做 2有些困难 3需要帮助 4根本无法做 |
| D12上厕所 | 1自己完全可以做 2有些困难 3需要帮助 4根本无法做 |
| D13打电话 | 1自己完全可以做 2有些困难 3需要帮助 4根本无法做 |
| D14处理自己钱物 | 1自己完全可以做 2有些困难 3需要帮助 4根本无法做 |

问卷12

**UCLA孤独感量表简版**

下面是人们有时出现的一些感受。对每项描述，请指出你具体有那种感觉的频度，将数字填在格内。

| 缺少别人的陪伴 | 1从不 | 2很少 | 3有时 | 4一直 |
| --- | --- | --- | --- | --- |
| 没有人可以寻求帮助 | 1从不 | 2很少 | 3有时 | 4一直 |
| 我感到被冷落 | 1从不 | 2很少 | 3有时 | 4一直 |
| 我感到和其他人疏远了 | 1从不 | 2很少 | 3有时 | 4一直 |
| 我因为很少与别人来往而感到伤心 | 1从不 | 2很少 | 3有时 | 4一直 |
| 虽然身边有人陪，但没人关心我 | 1从不 | 2很少 | 3有时 | 4一直 |

问卷13

**被访人情况**

被访人类别：1=第一被访人 2=第二被访人

被访人情况：

**若被访人为目标人本人，则结束基础问卷，跳至SCID。**

1.被访人与目标人的关系：

01=目标人本人 02=配偶 03=女朋友（恋爱关系） 04=男朋友（恋爱关系）

05=父亲 06=母亲 07=岳父 08=岳母

09=养父 10=养母 11=继父 12=继母

13=哥哥 14=弟弟 15=姐姐 16=妹妹

17=儿子 18=女儿 19=祖父 20=祖母

21=外祖父 22=外祖母 23=其他亲属 24=朋友

25=邻居 26=专业医疗人员 27=医疗/精神病记录

28=其他记录____________

2.婚姻状况：

01=从未结婚 02=已婚一起生活 03=已婚但分居

04=再婚 05=离异 06=鳏/寡居

07=未婚同居 08=其它____________

3.家庭人口（包括本人在内的同住人口）：_________________________

4.户口所在地（以身份证为准）：1=城市户口 2=农村户口

5.家庭年收入：_________________________元 99=不详

6.教育水平：上学年数_______________年

6a.最高学历：

1=小学 2=初中 3=高中/中专/高职 4=大专/大学 5=大学以上

7.宗教信仰

1=不信教 2=道教 3=穆斯林 4=基督教 5=天主教 6=佛教

7=其他_______________

8.若您有宗教信仰的话，平均每月能参加几次宗教活动？ ____________次/月。

9.您信不信神（迷信）？ 1=信 0=不信

10.您信不信人有来世？ 1=信 0=不信

11.您（当时-指目标人自杀以前）与他/她一起住吗？ 1=是 0=否

12.您最后一次见到目标人（在访谈/企图自杀/自杀前）有多长时间？ ________天

13.您最后一次与目标人谈话（在访谈/企图自杀/自杀前）有多长时间？______天

14.您（在访谈/目标人自杀前）一个月内见过目标人多少次？ ________次

15.您（在访谈/目标人自杀前）一个月内与目标人谈话有多少次？ ________次

16.您（在访谈/目标人自杀前）一年与目标人平均每月谈话有多少次？_______天

17.您（在访谈/目标人自杀前）认识目标人有多长时间？________年______月

18.总的来说，您觉得您对他/她的了解到程度如何？

1=十分了解 2=比较了解 3=一般了解 4=比较不了解 5=十分不了解

99=不详

19.根据您的了解，您觉得他/她自杀或以后会企图自杀的可能性有多大？

1=绝对可能 2=很有可能 3=有可能 4=不可能 99=不详

20.那么您对已经发生的事，怎样理解？

1=自杀 2=事故（如：车祸） 3=他杀 88=不适用 99=不详

21.根据您的判断，本村村民之间的关系紧密的程度如何？

1=很不紧密 2=不太紧密 3=一般 4=比较紧密 5=很紧密 99=不详

问卷14

**流行病调查中心抑郁量表（CESD）**

指导语：这部分是对您可能有过的或感到的情况和想法的描述，请按照过去一周内您的实际情况和感觉的频度选择合适的答案。

0=偶尔或无（少于1天） 1=有时（1-2天）

2=时常或一半时间（3-4天） 3=多数时间或持续（5-7天）

|  | 少于1天 | 1-2天 | 3-4天 | 5-7天 |
| --- | --- | --- | --- | --- |
| 1.一些通常并不困扰我的事使我烦恼。 | 0 | 1 | 2 | 3 |
| 2.我不大想吃东西，我胃口不好。 | 0 | 1 | 2 | 3 |
| 3.即使有家人或朋友帮助我，我仍然无法摆脱心中的苦闷。 | 0 | 1 | 2 | 3 |
| 4.我觉得我和其他人一样好。 | 0 | 1 | 2 | 3 |
| 5.我在做事时，无法集中自己的注意力。 | 0 | 1 | 2 | 3 |
| 6.我感到情绪低落。 | 0 | 1 | 2 | 3 |
| 7.我感到做任何事都很费力。 | 0 | 1 | 2 | 3 |
| 8.我感到前途是有希望的。 | 0 | 1 | 2 | 3 |
| 9.我感到我的生活是失败的。 | 0 | 1 | 2 | 3 |
| 10.我感到害怕。 | 0 | 1 | 2 | 3 |
| 11.我睡不好觉。 | 0 | 1 | 2 | 3 |
| 12.我很幸福。 | 0 | 1 | 2 | 3 |
| 13.我比平时说话要少。 | 0 | 1 | 2 | 3 |
| 14.我感到孤独。 | 0 | 1 | 2 | 3 |
| 15.我觉得人们对我不友好。 | 0 | 1 | 2 | 3 |
| 16.我觉得生活很有意思。 | 0 | 1 | 2 | 3 |
| 17.我曾失声哭泣。 | 0 | 1 | 2 | 3 |
| 18.我感到忧愁。 | 0 | 1 | 2 | 3 |
| 19.我感到人们不喜欢我。 | 0 | 1 | 2 | 3 |
| 20.我提不起精神来。 | 0 | 1 | 2 | 3 |

问卷15

**自杀意向、计划、姿态、企图量表**

1.您是否曾经认真地考虑过想死？

1=是 0=否

**如果该题答案为“否”，则跳过2-7，请直接到问卷13。**

2.在过去12个月中，您是否有过这种想法？

1=是 0=否

3.您是否曾经做过自杀的计划？

1=是 0=否

4.如果上题的答案为“是”，那么在过去12个月中，您是否做过这种计划？

1=是 0=否

5.您是否曾经有过自杀行为？

1=是 0=否

6.如果上题的答案为“是”，那么在过去12个月中，您是否有过自杀行为？

1=是 0=否

**如果该题答案为“否”，则跳过7，请直接到问卷13。**

7.下面三种陈述中，哪一种最好地描述了您的企图？

1.您确实要自杀，但只是运气不好，没有成功。

2.您试图自杀，但您知道那种办法不行。

3.您企图自杀是苛求关注或帮助，而不是想死。
