# Supplementary material for: Studying suicide using proxy-based data: reliability and validity of a short version scale for measuring quality of life in rural China
Source: PeerJ. 2021 Nov 9;9:e12396. doi: 10.7717/peerj.12396 (PMC8588864; doi:10.7717/peerj.12396)
Supplement: Supplemental Information 3 [file peerj-09-12396-s003.doc]

**Questionnaire number:** ________________________ **Interviewer’s name:**_________________________

**Target category:** 1=case group 2=control group

**Interviewee category:** 1=first informant 2=second informant 0=control group

**Target person's name:** _________ Birthday: ______year____month____day (Lunar/Gregorian calendar) Zodiac_________,

Age: ______ Gender: 1=Male 0=Female

**Interviewee's name:** _________ Birthday: ______year____month____day (Lunar/Gregorian calendar) Zodiac ______,

Age: ______ Gender: 1=Male 0=Female

**The relationship between the interviewee and the target person:** The interviewee is the target person's ____________

**Interviewee's home address:** ________ province ________ (city/county) ________ (township/town) ________ village, home phone number: ____________________

**Data collection method:** 1=face-to-face interview 2=phone 3=use both 4=historical data

**Interview date:** __________year__________month__________day

**Resident area of ​​the target person:** 1=rural 2=urban

**Interview location:** 1=Interviewee’s home 2=Hospital 3=Other locations ____________________ (please specify)

**The entire interview time (calculated on a 24-hour system):**

Starting time:__________:__________

End Time:__________:__________

**Total interview time:** ____________________ minutes

Questionnaire 1

**Case or control**

**Basic situation of the target:**

**1. His/her (your) household registration location:** 1=urban household registration 2=rural household registration

**2. His/her (your) education level:** number of school years __________ years

99=unknown

**3. Highest degree:**

1=illiterate 2=primary school 3=junior high school 4=high school/secondary school/higher vocational 5=junior college/university 6=university and above 88=not applicable 99=unknown

**4. His/her (your) marital status:**

1=Single, never married 2=Married living together 3=Married but separated

4=Remarried 5=Divorced 6=Widow/Widowhood

7=Unmarried living together 88=Other________ 99=Unknown

**5. Occupation:**

1=farmers/herders/fishers 2=self-employed 3=cadres (civil servants/managers) 4=Workers (including those who work in cities) 5=Students 6=Teachers 7= Village doctor 8=Other____________(please specify) 99=Unknown

**6. Labor or work conditions:**

1=Employed/self-employed 2=Unemployed 3=Retired 88=not applicable 99=unknown

**7. His/her (your) personal annual income** ________________ yuan 99=unknown

**8.His/her (your) family average annual income:** ________________ yuan 99=unknown

**9. His/her (your) family population** (residents including yourself): ________ people

**10. Whom does he/she (you) live with?** (Multiple choice)

Spouse/common-law spouse 1

Minor children of you or your spouse 2

Adult children of you or your spouse 3

Your grandchildren 4

You or your spouse’s parents 5

Other relatives 6

Others 7

Living Alone 8

11. Does he/she (you) have any children? If so, how many children are there? _______________ (please record in descending order of age)

| relationship | Age | marital status | Place of residence | Reason of residence | Frequency of home visits in the past year ("going home" refers to returning to your parents' home) | Total time spent living together in the past year |
| --- | --- | --- | --- | --- | --- | --- |
|  |  |  |  |  |  |  |
|  |  |  |  |  |  |  |
|  |  |  |  |  |  |  |
|  |  |  |  |  |  |  |
|  |  |  |  |  |  |  |
|  |  |  |  |  |  |  |
|  |  |  |  |  |  |  |
|  |  |  |  |  |  |  |
|  |  |  |  |  |  |  |
|  |  |  |  |  |  |  |

**Relationship code:** 1. Son 2. Daughter 3. Daughter-in-law 4. Son-in-law

**Marital status code:** 1. Married 2. Divorced 3. Widowed 4. Separated 5. Never married

**Residence code:** 1. Live together (a building or a yard) 2. The village 3. The township 4. Nearby, this county/city 5. This province 6. Other provinces/foreign countries

**The reason code of the place of residence:** 1. Live locally 2. Married (only for daughters) 3. Settled to study/work in another place (household registration has moved out) 4. Migrant for work (household registration is still local) 5. Others

**Frequency code of home visits in the past year:** 1. Live together 2. Once a week or more 3. 1-3 times/month 4. 3-11 times/year 5. 0-2 times/year

**Coding of total living together time:** 1.10-12 months 2.7-9 months 3.3-6 months 4.0-2 months

**12. What is his/her (you) status in the family?**

1=Highest 2=High 3=General 4=Low 5=Lowest 99=Unknown

**13. Religious belief**

1=Not religious 2=Taoism 3=Muslim 4=Christian 5=Catholic 6=Buddhism

7=other_____________ 99=unknown

**14. If he/she (you) has religious beliefs, how many religious activities can he/she (you) participate in on average every month?** ______ times/month

**15. Does he/she (you) believe in God (superstition)?** 1=Believe 0=Not Believe 99=Unknown

**16. Does he/she (you) believe that people have an afterlife?** 1=Believe 0=Not Believe 99=Unknown

**17. Is he/she (you) a member of the Communist Party?** 1=Yes 0=No 99=Unknown

**18. His/her (your) health status generally belongs to**:

1=very poor 2=relatively poor 3=average 4=fairly good 5=very good 99=unknown

**19. Has he/she (you) ever suffered from a serious or chronic disease** (referring to a disease that has been diagnosed in a medical institution)?

1=Yes 0=No 88=Not applicable 99=Unknown

If the answer is "No", please mark _ _____ here, skip 20-26, and go directly to 27.

**20. If yes, list the three most serious ones in order:** 1=_____________ 2=_____________ 3=_____________

**21. To what extent do these diseases affect his/her (you) daily life?**

1=No impact 2=Slight impact 3=Moderate impact 4=Deep impact 99=Unknown

**22. To what extent do these diseases affect his/her (you) mental state?**

1=No impact 2=Slight impact 3=Moderate impact 4=Deep impact 99=Unknown

**23. Has he/she (you) ever said that these diseases pose a threat to his/her (you) life or mentioned that this disease will not last long?**

1. Yes 2. No 99. Unknown

**24. Has he/she (you) said that these diseases bring financial burden to the family? If so, how serious is it?**

1. No burden at all 2. A relatively light burden 3. A relatively serious burden 4. A heavy burden

**25. Has he/she (you) ever said that these diseases need to be taken care of, which brings physical and mental burdens to the family?**

1. No burden at all 2. A relatively light burden 3. A relatively serious burden 4. A heavy burden

**26. Did he/she (you) feel any pain (including pain caused by various physical diseases or unexplained pain) before the accident (recently)? If so, how many points can be scored? 0 is no pain at all, and 10 is the most severe unbearable pain.**

|  |  |  |  |  |  |  |  |  |  |
| --- | --- | --- | --- | --- | --- | --- | --- | --- | --- |

0 1 2 3 4 5 6 7 8 9 10

**27. Does he/she (you) have a mental illness?** 1=Yes 0=No 99=Unknown

**28. If "Yes", has he/she (you) been treated?** 1=Yes 0=No 99=Unknown

**29. Has his/her (your) family member (note: refers to blood related) ever committed suicide (note: including suicide attempt and suicide death)?**

1=Yes 0=No 99=Unknown

If yes, this family member is ______________; the method of suicide is ____________________

**30. Has any of his/her (your) other unrelated family members or acquaintances committed suicide (note: including suicide attempts and suicide deaths)?**

1=Yes 0=No 99=Unknown

If so, this person is __________________________; his suicide method is ____________________

**31. Is there any pesticide in his/her (your) home?** 1=Yes 0=No 99=Unknown

If yes, please list the three main pesticide names:

**1_______________ 2_______________ 3_______________**

32-36 is suitable for the target person in the suicide death group and the target person in the control group. If it is the target person in the control group, the hospital (clinic) is the nearest hospital (clinic) to the target person in the control group, and the distance is the distance from the home to that hospital (clinic).

**32. He/she was sent to (the nearest to your home) ______________________ hospital (clinic) after the accident; the location is _______________; its level is: _______________**

**33. The official name of his/her (your) village:** _______________.

**34. The estimated distance from the place of origin (your home) to the hospital:** __________.

**35. The time it takes to go to the hospital from the place of origin** (your home): __________ hours.

**36. Before the accident/in the last week or month, did he/she see a doctor?**

1=Yes 0=No 99=Unknown

|  | Past a week | Past a month |
| --- | --- | --- |
| Chinese Medicine |  |  |
| Psychiatrist or other mental health worker |  |  |
| Village doctor |  |  |
| Other doctors in township health centers |  |  |
| Other doctors in hospitals at or above the county level |  |  |

Questionnaire 3

**Suicidal behavior**

Now I want to ask you some details related to his/her (attempted) suicide and the event that triggered this event. Please recall the details as much as possible. (Note: If the target person is the target person in the control group, skip directly to question 21.)

**1. Date of his/her suicide (non-death time):** ________year______month______day

**2. The exact time when his/her suicide occurred** (recorded in a 24-hour way): ______hour______minute

**3. Which method did the suicide person use first**: __________

1=Drink pesticides 2=Drink other poisons 3=Hanging 4=Throwing into the river 5=Jumping 6=Overdose 7=Cut wrist 8=gas 9=suffocation 10=gun 11=electric shock 12=sleeping on rail 13=other________ 88=not applicable 99=unknown

**4. The method leading to the death of a suicide is:** ____________________

**5. The place where he/she committed suicide is** 1=home 2=other places ________________________

**6. Did he/she drink alcohol within 6 hours before committing suicide?** 1=Yes 0=No 99=Unknown

If you choose "Yes":

(1) How long did he/she drink alcohol before committing suicide? ___________________hour

(2) How many "cups" did he/she drink before committing suicide? (One or two high liquor = one bottle of beer = 2 glasses; if it is a private brew, such as rice wine or sorghum wine, please record the degree and amount)

__________________ glass or ___________ two___________ degrees of wine

**7. Where was he/she when it happened? Who else is there or nearby?**

(SIS-1) Isolation

0=Someone is present (refers to face-to-face)

1=Someone is nearby, can see or hear

2=No one nearby, or no one sees or hears

**8. What did he/she do to prevent being discovered? (If you lock the door, choose an unfamiliar place, have clues and hints) Who found him/her? How did you find it?**

(SIS-2) In terms of time

0=may be blocked

1=Not likely to be blocked

2=It is completely impossible to be blocked

**9. (SIS-3) Prevention (Explanation: Be prepared in advance to prevent the behavior from being discovered)**

0=No protection

1=Passive prevention (such as avoiding others, but not preventing others from interfering; the door of the room alone is not locked)

2=Active prevention (such as locking the door)

**10. (SIS-4) Ask for help**

0=Telled suicide attempt to someone who might help oneself

1=Contacted someone who might help you but did not clearly indicate a suicide attempt

2=Have not contacted someone who might help you or informed you of the suicide attempt

**11. Has he/she made any preparations for death? (If you wish, or give away something that has special meaning to you?)**

(SIS-5) Funeral arrangements

0=no

1=Considered or made some arrangements

2=Make a definite plan or complete the arrangement

**12. How much has he/she planned for suicide? (Such as cutting veins or searching for poisons, storing pills, etc.)**

(SIS-6) Actively prepare

0=No preparation

1=partial/light to moderate preparation

2=fully/fully prepared

**13. Did he/she write a last word or suicide note, or write a suicide plan in the diary? Does he/she plan to do this?**

(SIS-7) Last words, suicide notes or related diaries

0=no

1=Writing last words, suicide notes but torn apart, or mentioned in diaries but crossed out; last words, letters or diaries considering suicide

2=There are last words, suicide notes or related diaries

**14. Apart from leaving a message, who did he/she tell about his plan? What is being said? ____________ Has ever said anything that would say "I should have known that he would commit suicide" when it reminded others?**

(SIS-8) The suicide attempt was clearly mentioned before the action

0=no

1=Vaguely mentioned

2=Explicitly mentioned

**15. After suicide, who first discovered him/her?**

1=family member 2=relative 3=friend 4=other 99=unknown

**16. After discovering that he/she committed suicide, what was the witness's first reaction?**

1=Call the emergency number 2=Tell the family of the target person 3=Call the doctor 4=other 99=unknown

**17. After he/she was found, was he/she sent to the hospital immediately?**

1=Yes 0=No 99=Unknown

**18. After the incident, did anyone immediately rescue him/her?**

1=Yes 0=No 99=Unknown

**19. How long does it take to receive effective treatment after he/she is discovered?** _______________hour

**20. Do you think the direct reason that triggered his/her suicide is**: _________________________

**21. Has he/she (you) ever tried to commit suicide? The number of attempts to commit suicide in the past**: ____________

If the answer is 0 times, please go directly to the next questionnaire.

**22. How long is it (now) since the last attempt to commit suicide?**

1=1~7 days 2=2~4 weeks 3=2~6 months 4=7~12 months 5=2 years

6=3~5 years 7=6 years and above 88=not applicable 99=unknown

**23. The last attempt to commit suicide was**:

1=Drink pesticides 2=Drink other poisons 3=Hanging 4=Throwing into the river 5=Jumping 6=Overdose 7=cut wrist 8=gas 9=suffocation 10=gun 11=electric shock 12=sleeping on rail 13=Other________ 88=Not applicable 99=Unknown

Questionnaire 4

**BIS Impulse Scale Chinese Version**

In different situations, people's behavior and thinking are different. This is a scale to measure some of your behaviors and ways of thinking. Please read it carefully and answer quickly and truthfully.

| 1. I arrange everything carefully. | No | Rarely | Sometimes | Often | always |
| --- | --- | --- | --- | --- | --- |
| 2. I do things without thinking. | No | Rarely | Sometimes | Often | always |
| 3. I can think of a good way when I encounter a problem. | No | Rarely | Sometimes | Often | always |
| 4. I have plans for the future. | No | Rarely | Sometimes | Often | always |
| 5. I can't control my behavior well. | No | Rarely | Sometimes | Often | always |
| 6. I can think about a problem for a long time if necessary. | No | Rarely | Sometimes | Often | always |
| 7. I save or save money regularly. | No | Rarely | Sometimes | Often | always |
| 8. I can't control my temper. | No | Rarely | Sometimes | Often | always |
| 9. I can consider issues from different angles. | No | Rarely | Sometimes | Often | always |
| 10. I have a plan for work and income. | No | Rarely | Sometimes | Often | always |
| 11. I speak without thinking. | No | Rarely | Sometimes | Often | always |
| 12. I like to think slowly when I encounter problems. | No | Rarely | Sometimes | Often | always |
| 13. I do things more rationally. | No | Rarely | Sometimes | Often | always |
| 14. It is difficult to control my behavior when I am excited. | No | Rarely | Sometimes | Often | always |
| 15. When encountering a problem, I can patiently think about solutions to the problem. | No | Rarely | Sometimes | Often | always |
| 16. I regularly arrange my diet and daily life. | No | Rarely | Sometimes | Often | always |
| 17. I tend to act impulsively. | No | Rarely | Sometimes | Often | always |
| 18. Before making a decision, I like to carefully consider the gains and losses. | No | Rarely | Sometimes | Often | always |
| 19. I arrange everything before I leave home. | No | Rarely | Sometimes | Often | always |
| 20. I act immediately without considering the consequences. | No | Rarely | Sometimes | Often | always |
| 21. I think about the problem calmly. | No | Rarely | Sometimes | Often | always |
| 22. When I do things, I can finish as planned. | No | Rarely | Sometimes | Often | always |
| 23. I tend to shop impulsively. | No | Rarely | Sometimes | Often | always |
| 24. I will not easily draw conclusions when encountering problems. | No | Rarely | Sometimes | Often | always |
| 25. I spend money in a planned way. | No | Rarely | Sometimes | Often | always |
| 26. I act recklessly. | No | Rarely | Sometimes | Often | always |
| 27. I can concentrate when thinking about problems. | No | Rarely | Sometimes | Often | always |
| 28. I attach great importance to the arrangements for the future. | No | Rarely | Sometimes | Often | always |
| 29. I do what I think of immediately. | No | Rarely | Sometimes | Often | always |
| 30. It is easy for me to come up with new ways to solve the difficulties encountered. | No | Rarely | Sometimes | Often | always |

Questionnaire 5

**Despair Scale**

Instruction:

Everyone has some views on the future, what is the target person’s view? Each of the following questions has 5 answers:

1. Totally Yes 2. Almost Yes 3. It's hard to be sure. 4. Basically contrary. 5. Totally contrary.

Please choose the most suitable answer based on the situation of the target person one week before the suicide (one week before the interview).

|  | Totally Yes | Almost Yes | It's hard to be sure. | Basically contrary | Totally contrary |
| --- | --- | --- | --- | --- | --- |
| 1. He/she (you) hope to do the most important thing well in the future. | 1 | 2 | 3 | 4 | 5 |
| 2. His/her (your) future is dark. | 1 | 2 | 3 | 4 | 5 |
| 3. He/she (you) is out of luck, and does not expect to be able to operate from time to time in the future. | 1 | 2 | 3 | 4 | 5 |
| 4. He/she (you) is full of confidence in the future. | 1 | 2 | 3 | 4 | 5 |

Questionnaire 6

**Quality of life**

Next, I would like to know your quality of life in the last month before the suicide or investigation.

| How was your physical health in the last month? | excellent | good | fair | poor | very poor |
| --- | --- | --- | --- | --- | --- |
| How was your psychological health in the last month? | excellent | good | fair | poor | very poor |
| How was your economic status in the last month? | excellent | good | fair | poor | very poor |
| How was your work (study or farm work) in the last month? | excellent | good | fair | poor | very poor |
| How were your relationships with your family in the last month? | excellent | good | fair | poor | very poor |
| How were your relationships with others in the last month? | excellent | good | fair | poor | very poor |

Questionnaire 7

**Elderly Life Event Scale**

Guidance: The following table lists events that may occur in life. Each event has five columns, which are the time and nature of the event, the degree of psychological impact, the duration of the impact, and the number of occurrences of the event. Please select the most appropriate item to mark "√" in each column. If the event did not occur, just underline “√” in the item “not occurred”, and leave the remaining four columns blank.

| **Life events** | **Time of occurrence** | | | **nature** | | **Psychological impact** | | | | | **Duration of impact** | | | | **Frequency of occurrence** |
| --- | --- | --- | --- | --- | --- | --- | --- | --- | --- | --- | --- | --- | --- | --- | --- |
|  | There was no | Within a year | long-  term | Good | bad | no effect | Mild | Moderate | Severe | Extremely heavy | Within three months | Half a year | One year | More than one year |  |
| Example: Moving |  | √ |  |  | √ |  | √ |  |  |  |  | √ |  |  | 1 |
| Health-related issues |  |  |  |  |  |  |  |  |  |  |  |  |  |  |  |
| 1.Suffer from chronic diseases |  |  |  |  |  |  |  |  |  |  |  |  |  |  |  |
| 2. I am seriously ill or terminally ill |  |  |  |  |  |  |  |  |  |  |  |  |  |  |  |
| 3. Family members are seriously ill or terminally ill |  |  |  |  |  |  |  |  |  |  |  |  |  |  |  |
| 4. I was injured in a traffic accident or other accident |  |  |  |  |  |  |  |  |  |  |  |  |  |  |  |
| 5. Family members are injured due to traffic accidents or other accidents |  |  |  |  |  |  |  |  |  |  |  |  |  |  |  |
| 6. I am hospitalized |  |  |  |  |  |  |  |  |  |  |  |  |  |  |  |
| 7. Family members are hospitalized |  |  |  |  |  |  |  |  |  |  |  |  |  |  |  |
| 8. I take care of my own difficulties |  |  |  |  |  |  |  |  |  |  |  |  |  |  |  |
| 9. Family members take care of their own life difficulties |  |  |  |  |  |  |  |  |  |  |  |  |  |  |  |
| 10. I have recovered from my illness |  |  |  |  |  |  |  |  |  |  |  |  |  |  |  |
| 11. Family members recover from illness |  |  |  |  |  |  |  |  |  |  |  |  |  |  |  |
| 12. Relatives or friends are seriously ill |  |  |  |  |  |  |  |  |  |  |  |  |  |  |  |
| 13. Death of spouse |  |  |  |  |  |  |  |  |  |  |  |  |  |  |  |
| 14. Child death |  |  |  |  |  |  |  |  |  |  |  |  |  |  |  |
| 15. The child's spouse dies |  |  |  |  |  |  |  |  |  |  |  |  |  |  |  |
| 16. Death of a relative or friend |  |  |  |  |  |  |  |  |  |  |  |  |  |  |  |
| Family life related issues: |  |  |  |  |  |  |  |  |  |  |  |  |  |  |  |
| 17. Intense quarrel or fight with spouse |  |  |  |  |  |  |  |  |  |  |  |  |  |  |  |
| 18. Separation of husband and wife |  |  |  |  |  |  |  |  |  |  |  |  |  |  |  |
| 19. Divorce |  |  |  |  |  |  |  |  |  |  |  |  |  |  |  |
| 20. I have an affair |  |  |  |  |  |  |  |  |  |  |  |  |  |  |  |
| 21. Spouse has an affair |  |  |  |  |  |  |  |  |  |  |  |  |  |  |  |
| 22. Husband and wife get back together |  |  |  |  |  |  |  |  |  |  |  |  |  |  |  |
| 23. Children have intense quarrels with their spouses |  |  |  |  |  |  |  |  |  |  |  |  |  |  |  |
| 24. Family financial difficulties |  |  |  |  |  |  |  |  |  |  |  |  |  |  |  |
| 25. Living alone |  |  |  |  |  |  |  |  |  |  |  |  |  |  |  |
| 26. Crowded housing |  |  |  |  |  |  |  |  |  |  |  |  |  |  |  |
| 27. Theft or major loss of house or property |  |  |  |  |  |  |  |  |  |  |  |  |  |  |  |
| 28. Children leave home for a long time |  |  |  |  |  |  |  |  |  |  |  |  |  |  |  |
| 29. Children are not filial |  |  |  |  |  |  |  |  |  |  |  |  |  |  |  |
| 30. Major changes in eating or sleeping habits |  |  |  |  |  |  |  |  |  |  |  |  |  |  |  |
| 31. Discord between family members |  |  |  |  |  |  |  |  |  |  |  |  |  |  |  |
| 32. Children who are laid off or have difficulty finding jobs |  |  |  |  |  |  |  |  |  |  |  |  |  |  |  |
| 33. Personal residence or living conditions have changed significantly |  |  |  |  |  |  |  |  |  |  |  |  |  |  |  |
| 34. The economic situation has improved significantly |  |  |  |  |  |  |  |  |  |  |  |  |  |  |  |
| Social and other issues |  |  |  |  |  |  |  |  |  |  |  |  |  |  |  |
| 35. Tension or dispute with neighbors |  |  |  |  |  |  |  |  |  |  |  |  |  |  |  |
| 36. Separate or break with friends |  |  |  |  |  |  |  |  |  |  |  |  |  |  |  |
| 37. No close friends, loneliness |  |  |  |  |  |  |  |  |  |  |  |  |  |  |  |
| 38. I resign and retire |  |  |  |  |  |  |  |  |  |  |  |  |  |  |  |
| 39. Spouse resigns and retire |  |  |  |  |  |  |  |  |  |  |  |  |  |  |  |
| 40. I am involved in a legal dispute |  |  |  |  |  |  |  |  |  |  |  |  |  |  |  |
| 41. Family members involved in legal disputes |  |  |  |  |  |  |  |  |  |  |  |  |  |  |  |
| 42. I have suffered face loss and discrimination |  |  |  |  |  |  |  |  |  |  |  |  |  |  |  |
| 43. Misunderstood or blamed |  |  |  |  |  |  |  |  |  |  |  |  |  |  |  |
| 44. I have been intimidated or beaten by others |  |  |  |  |  |  |  |  |  |  |  |  |  |  |  |
| 45. Family members are threatened or beaten by others |  |  |  |  |  |  |  |  |  |  |  |  |  |  |  |
| 46. ​​Being deceived by others |  |  |  |  |  |  |  |  |  |  |  |  |  |  |  |
| If you have experienced other life events in the last year, please fill in |  |  |  |  |  |  |  |  |  |  |  |  |  |  |  |
|  |  |  |  |  |  |  |  |  |  |  |  |  |  |  |  |
|  |  |  |  |  |  |  |  |  |  |  |  |  |  |  |  |

Questionnaire 8

Duke Social support scale (DSSI)

Social interaction subscale

1. In addition to family members, within an hour's reach in this area, how many people he/she can rely on or feel close to?

1=0 2=1~4 people 3=5 people or more

1. In addition to work, how many times did he/she interact with people who do not live together in the week before the suicide (you were in the previous week): including him/her going to see others, others visiting him/her or them ( You) go out together.

1=0 2=1~4 people 3=5 people or more

1. In addition to work, how many times did he/she talk to friends, relatives or other people on the phone in the week before suicide (you were in the last week) (calling him/her or him/her to others is counted) ).

1=0 2=1~4 times 3=5 times or more

1. In addition to work, how many times did he/she attend various gatherings in the week before suicide (you were in the previous week)?

1=0 2=1~4 times 3=5 times or more

Subjective social support

5. Does he/she say that his/her family and friends (i.e. the people who are important to him/her) understand him/her?

1= Almost never said it 2= Said it sometimes 3= Said it often

6. Has he/she ever said he/she was useful to family and friends (i.e., the people who matter to him/her)?

1= Almost never said it 2= Said it sometimes 3= Said it often

7. Does he/she say he/she knows how his/her family and friends are doing?

1= Almost never said it 2= Said it sometimes 3= Said it often

8. Does he/she say that he/she feels listened to when talking to family and friends?

1= Almost never said it 2= Said it sometimes 3= Said it often

9. Does he/she say that he/she has a defined place (role) among family and friends?

1= Almost never said it 2= Said it sometimes 3= Said it often

10. Does he/she say that he/she can confide in at least some of his/her family and friends?

1= Almost never said it 2= Said it sometimes 3= Said it often

11. Does he/she say how satisfied he/she is with his/her family and friends? If he/she does not have family and friends, is he/she satisfied with not having such relationships?

1= very dissatisfied 2= somewhat satisfied 3= satisfied

**Social support utilization**

Now I want to ask some family members and friends he/she perceives to help him/her. Has he/she ever said that his/her family has helped him/her in the following ways?

12. Help him/her when he/she is sick?

1=Yes 0=No

13. Help him/her do shopping or run errands?

1=Yes 0=No

14. Give him/her a gift?

1=Yes 0=No

15. Spend money to help him/her?

1=Yes 0=No

16. Help him/her clean up the surroundings of the house?

1=Yes 0=No

17. Help him/her tidy up the house and do housework?

1=Yes 0=No

18. Advise him/her on business or financial issues?

1=Yes 0=No

19. Accompany him/her?

1=Yes 0=No

20. Listen to him/her complaining?

1=Yes 0=No

21. Advise him/her on how to deal with life problems?

1=Yes 0=No

22. Provide convenient transportation for him/her?

1=Yes 0=No

23. Cook for him/her or invite him/her to eat?

1=Yes 0=No

Questionnaire 9

Geriatric depression scale

Note: Please choose the answer that best matches your feelings during the past week. After the investigator finishes reading a sentence, answer with a yes or no.

| 1. Are you generally satisfied with your life? | Yes | No |
| --- | --- | --- |
| 2. Have you given up many activities and interests? | Yes | No |
| 3. Do you feel that life is empty? | Yes | No |
| 4. Are you bored? | Yes | No |
| 5. Do you see any hope for the future? | Yes | No |
| 6. Are you upset because you can't get rid of some ideas in your mind? | Yes | No |
| 7. Are you energetic most of the time? | Yes | No |
| 8. Are you afraid that unfortunate things will fall on you? | Yes | No |
| 9. Do you feel happy most of the time? | Yes | No |
| 10. Do you often feel isolated? | Yes | No |
| 11. Do you often fidget and get upset? | Yes | No |
| 12. Are you willing to stay at home rather than do something new? | Yes | No |
| 13. Do you often worry about the future? | Yes | No |
| 14. Do you think your memory is worse than before? | Yes | No |
| 15. Do you feel comfortable being alive now? | Yes | No |
| 16. Do you often feel heavy and depressed? | Yes | No |
| 17. Do you find it meaningless to live like this? | Yes | No |
| 18. Do you always worry about the past? | Yes | No |
| 19. Do you think life is very exciting? | Yes | No |
| 20. Is it difficult for you to start a new job? | Yes | No |
| 21. Do you think life is full of vitality? | Yes | No |
| 22. Do you feel that your situation is hopeless? | Yes | No |
| 23. Do you think most people are much better than you? | Yes | No |
| 24. Do you often feel sad for some small things? | Yes | No |
| 25. Do you often feel like crying? | Yes | No |
| 26. Do you have any trouble for concentrating? | Yes | No |
| 27. Do you enjoy getting up in the morning? | Yes | No |
| 28. Do you want to avoid parties? | Yes | No |
| 29. Is it easy for you to make a decision? | Yes | No |
| 30. Is your mind as clear as usual? | Yes | No |

Questionnaire 10

Family Function Questionnaire

| 1.When I encounter difficulties, I can get satisfactory help from my family | Often | sometimes | Rarely |
| --- | --- | --- | --- |
| 2. I am very satisfied with the way my family discusses various things with me and how they share problems | Often | sometimes | Rarely |
| 3. What I do can be accepted and supported by my family | Often | sometimes | Rarely |
| 1. I am very satisfied with the expression of mutual respect, acceptance, and value from my family | Often | sometimes | Rarely |
| 1. I am satisfied with the way my family spends time with me (travel, entertainment, etc.) | Often | sometimes | Rarely |

Questionnaire 11

Activities of daily living Scale

| **Test categories** | **score** |
| --- | --- |
| D1 Use of Public Vehicles | 1. I can do it by myself 2. I have some difficulties 3. I need help 4. I can't do it at all |
| D2 walking | 1. I can do it by myself 2. I have some difficulties 3. I need help 4. I can't do it at all |
| D3 Cooking | 1. I can do it by myself 2. I have some difficulties 3. I need help 4. I can't do it at all |
| D4 Do housework | 1. I can do it by myself 2. I have some difficulties 3. I need help 4. I can't do it at all |
| D5 Take medicine | 1. I can do it by myself 2. I have some difficulties 3. I need help 4. I can't do it at all |
| D6 Have a meal | 1. I can do it by myself 2. I have some difficulties 3. I need help 4. I can't do it at all |
| D7 Dress | 1. I can do it by myself 2. I have some difficulties 3. I need help 4. I can't do it at all |
| D8 Comb hair, brush teeth, etc. | 1. I can do it by myself 2. I have some difficulties 3. I need help 4. I can't do it at all |
| D9 wash clothes | 1. I can do it by myself 2. I have some difficulties 3. I need help 4. I can't do it at all |
| D10 Take a shower | 1. I can do it by myself 2. I have some difficulties 3. I need help 4. I can't do it at all |
| D11 Shopping | 1. I can do it by myself 2. I have some difficulties 3. I need help 4. I can't do it at all |
| D12 Go to the toilet | 1. I can do it by myself 2. I have some difficulties 3. I need help 4. I can't do it at all |
| D13 Make a phone call | 1. I can do it by myself 2. I have some difficulties 3. I need help 4. I can't do it at all |
| D14 Deal with your money | 1. I can do it by myself 2. I have some difficulties 3. I need help 4. I can't do it at all |

Questionnaire 12

Short version of UCLA Loneliness Scale

Here are some of the feelings that people sometimes experience. For each description, please indicate how often you feel that way, and fill in the number in the box.

| Lack of companionship | 1. Never | 1. Rarely | 1. Sometimes | 1. Always |
| --- | --- | --- | --- | --- |
| No one can ask for help | 1. Never | 1. Rarely | 3. Sometimes | 4. Always |
| I feel left out | 1. Never | 1. Rarely | 3. Sometimes | 4. Always |
| I felt alienated from the others | 1. Never | 1. Rarely | 3. Sometimes | 4. Always |
| I feel sad because I rarely interact with others | 1. Never | 1. Rarely | 3. Sometimes | 4. Always |
| Although there are people around, but no one cares about me | 1. Never | 1. Rarely | 3. Sometimes | 4. Always |
